# Cumulative disaster exposure and hypertension among mothers who survived Hurricane Katrina

**DOI:** 10.1088/2752-5309/adb32c

**Published:** 2025-02-17

**Authors:** Marie-Claire Meadows, Mayur M Desai, Meghan Zacher, Sarah R Lowe

**Affiliations:** 1 Division of Environmental Health Sciences, University of Minnesota School of Public Health, Minneapolis, MN, United States of America; 2 Department of Chronic Disease Epidemiology, Yale School of Public Health, New Haven, CT, United States of America; 3 Population Studies and Training Center, Brown University, Providence, RI, United States of America; 4 Department of Social and Behavioral Sciences, Yale School of Public Health, New Haven, CT, United States of America

**Keywords:** Hurricanes, hypertension, mental health

## Abstract

As climate change intensifies, hurricanes and weather-related disasters have been increasingly frequent and severe, impacting regions like the U.S. Gulf Coast with repeated hurricanes. While acute and short-term health impacts are well-described, impacts on longer-term and chronic conditions such as hypertension remain underexplored. This study examines the association between repeated hurricane exposure and hypertension risk in survivors. We used data from the Resilience in Survivors of Katrina project, a longitudinal (2003–2018) cohort of predominantly Black, low-income mothers affected by Hurricane Katrina. A sample of 505 women who were not hypertensive pre-Katrina was analyzed. Cumulative exposure was defined as the number of hurricanes experienced post-Katrina, assessed at several survey waves over 12 years. Logistic regression estimated associations between hurricane exposure and hypertension in 2016–18, with mediation analyses exploring the indirect effect via psychological distress (PD). In adjusted models, exposure to two hurricanes was associated with a 61% increase in hypertension odds (OR = 1.61, 95% CI: 1.00, 2.63) and exposure to three or more with 87% increased odds (OR = 1.87, 95% CI: 1.01, 3.47), relative to exposure to only one hurricane. The indirect effect from hurricane exposure to hypertension via PD was statically significant (95% CI: 1.01, 1.09). Findings highlight a novel link between cumulative disaster exposure and hypertension, with PD as a potential mediator. This suggests that repeated exposure to hurricanes not only impacts mental health but may also contribute to adverse physical health outcomes. Addressing both mental and physical health in disaster response, especially for vulnerable populations, is crucial.

## 1. Introduction

Hurricanes and other weather-related disasters are increasing in frequency and intensity as a result of climate change [1]. The United States’ Gulf Coast is particularly vulnerable, both geographically and socially, to these disasters, as evidenced by the number of major hurricanes that the area has experienced. One such disaster, Hurricane Katrina, which made landfall in 2005, caused over 1000 deaths and $108 billion in damage [2, 3]. The hurricane’s short- and longer-term impact on residents of the Gulf Coast has been thoroughly studied [4–6], with ample scholarship available on acute and short-term health outcomes [4, 7, 8]. However, post-disaster impacts on chronic conditions have not been studied as robustly, especially in the longer-term aftermath of disasters [9]. In particular, few studies have explored hypertension in groups that have experienced multiple disasters [10–12].

Hypertension, often referred to as high blood pressure, is defined as systolic blood pressure over 140 mmHg and diastolic blood pressure over 90 mmHg [13–15]. Genetic, environmental, and lifestyle factors contribute to this ‘silent killer’, which commonly shows no signs or symptoms and can be difficult to diagnose without regular screening [15]. Additionally, hypertension has been suspected of being a psychosomatic disease, with research indicating that psychological conditions contribute to its development [13–15]. Ample research suggests that veterans and others who have experienced traumatic events have a higher likelihood of developing hypertension [10, 14, 16]. Relatedly, psychological distress (PD) has long been linked to hypertension outcomes [17]. Importantly, exposure to disasters, including hurricanes, can involve traumatic experiences and can increase PD [4]. It is therefore plausible that survivors of multiple hurricanes may be at increased risk for hypertension.

A small body of literature has shown the impact of weather-related disasters on hypertension outcomes. For example, a systematic review conducted in 2020 identified 13 articles that looked at hypertension after disasters and found that hypertension prevalence was greater among disaster survivors, compared to the general population [11]. Similarly, longitudinal work related to the Deepwater Horizon oil spill in the Gulf of Mexico and 11th September attacks in New York City have found that those exposed to these disasters have reported increased hypertension diagnoses [18–20]. In the broader trauma literature, cumulative exposure to adverse childhood experiences has been linked to a higher risk of cardiovascular disease [21]. However, no study has examined whether repeated exposure to disasters is associated with hypertension.

To address this gap in the literature, this study examined the association between cumulative hurricane exposure and hypertension, assessed 12 years later. We draw on several waves of data collected between 2003 and 2018 from a prospective cohort of primarily Black mothers, all of whom experienced Hurricane Katrina in 2005, and many of whom experienced additional hurricanes over the following 12 years. Using these data, we sought to identify relationships between their experiences and physical health.

## 2. Methods

Data were from the Resilience in Survivors of Katrina (RISK) project. Initially called the Opening Doors Study, which investigated an intervention to improve retention at community colleges, the RISK project enrolled individuals between the ages of 18 and 34 who were parents and earned less than 200% of the federal poverty line at the time of enrollment in 2003 (*n* = 1019). This cohort was largely composed of non-Hispanic Black, unmarried mothers, who were surveyed several times since enrollment [22].

Participants completed the study’s first survey between November 2003 and February 2005, before Hurricane Katrina (Baseline). Since Katrina, three surveys have been completed: March 2006–March 2007 (PK1); March 2009–April 2010 (PK2); and November 2016–December 2018 (PK3). For each survey, greater than 70% of participants from the original cohort responded. Although men were eligible for participation at baseline, few were recruited, therefore, only respondents identifying as female were re-surveyed.

The sample for this study was restricted to those who responded to PK3, when hypertension was assessed. From the 716 women who responded to PK3, we excluded 10 who reported a hypertension diagnosis prior to Katrina or at baseline. Additionally, 18 respondents were excluded due to missing hypertension data at PK3 and 183 were excluded due to missing data for covariates. The final sample therefore included 505 women without hypertension prior to Katrina and with complete data on hypertension status at PK3 and covariates.

## 3. Measures

### 3.1. Hypertension

Hypertension data was used from Baseline and PK3, as participants were not explicitly asked about hypertension status at PK1 or 2. Respondents were asked to self-report whether they had been diagnosed with hypertension by a physician. Hypertension was coded as a binary variable (0 = never diagnosed with hypertension; 1 = diagnosed with hypertension).

### 3.2. Hurricane exposure

At each post-Katrina survey wave, participants were asked whether they had experienced hurricanes that had made landfall in the United States or U.S. territories since the previous survey wave. Hurricanes were not limited to those that had affected the initial study area of New Orleans, Louisiana, as several participants have moved from the area since Katrina. Hurricanes included in the total number experienced were Hurricanes Katrina (2005), Rita (2005), Gustav (2008), Ike (2008), Irene (2011), Sandy (2012), Isaac (2012), Harvey (2017), Irma (2017), and Maria (2017). Cumulative exposure was the sum of all affirmative responses to questions asking if the participant had experienced the specific hurricane. Responses in this sample ranged from 1 to 8.

### 3.3. Covariates

Sociodemographic, Katrina-related trauma exposures, psychological outcomes, and clinical characteristic covariates were used for this study. Specifically, the variables used were self-reported race/ethnicity, age at PK3, marital status at PK3, a count of hurricane-related traumas from Katrina, PD at PK2, BMI at PK3, and self-perceived overall health at PK3. Race and marital status were coded as binary variables (0 = other race; 1 = Non-Hispanic Black) and (0 = not married; 1 = married), respectively. Age was assessed continuously. To control for the effects of Katrina, Hurricane Katrina-related traumas were constructed based on previous literature [23] as the sum of affirmative responses to several potentially traumatic experiences that happened during or just after the storm: believed life was in danger, could not access medications, could not access medical care, did not know if child was safe, did not know if another relative was safe, had a relative who could not access medical care, lacked sufficient food, and neighborhood flooded. PD was assessed using the six-item Kessler-6 scale at baseline [24]. Participants were asked to indicate on a 4-item scale from none of the time to all of the time how often in the previous 30 d they experienced feelings related to PD, such as ‘hopeless’ and ‘worthless’. In the current sample, Cronbach’s alpha value for internal consistency was 0.75. BMI was split into three categories: less than 25 kg m^−2^, between 25 kg m^−2^ and 29.9 kg m^−2^, and 30 kg m^−2^ or higher, based on self-reported height/weight [25]. Self-perceived general health at baseline was assessed using a Likert- type scale, excellent, very good, good, fair, and poor. A three-level variable was then created, combining very good and good, and fair and poor answers [26].

## 4. Analysis

A description of the sample was calculated, including means, standard deviations (continuous variables), and percentages (categorical variables). To assess the relationship between hypertension and cumulative hurricane exposure, logistic regression was used. First, unadjusted associations were calculated, followed by adjusted associations using all covariates in the models. Finally, to assess the role played by psychological conditions such as PD on the relationship between hurricane experience and hypertension, a mediation analysis was performed. The indirect effect of hurricane exposure on hypertension via PD was estimated using a mediation analysis based on a two-step linear regression model approach [27]. To quantify the uncertainty of the indirect effect, bootstrapping with 1000 simulations was used to generate a 95% confidence interval. A confidence interval that did not include zero was considered statistically significant. All analyses were conducted in R Statistical Software (version 4.2.2), with mediation analyses conducted using the mediation R package [28, 29].

## 5. Results

Those with hypertension had a mean age of 26.0 years (SD = 4.4), slightly older than both those without hypertension (24.6 years, SD = 4.4), and the full sample (25.1 years, SD = 4.4) (table 1). Participants with hypertension experienced on average 3.0 traumatic events during or immediately after Katrina, compared to 2.9 among those without hypertension. Individuals with hypertension also scored higher on average on the Kessler-6 scale (5.3) than those without (4.9). Additionally, 42.2% of hypertensive participants reported an obese BMI, compared to 22% of non-hypertensive participants. Finally, those with hypertension were more likely to experience more hurricanes, with 13.8% seeing three or more hurricanes compared to only 8.7% in the non-hypertensive group.

**Table 1.** Description of the sample by hypertension status.

Characteristic	Total Cohort *N* (%) Mean ± SD (*N* = 505)	Hypertension status	
		No hypertension (*N* = 332)	Hypertension (*N* = 173)
**Hurricanes experienced**			
1	356 (70.5)	247 (74.4)	109 (63.0)
2	96 (19.0)	56 (16.9)	40 (23.1)
⩾3	53 (10.5)	29 (8.7)	24 (13.8)
**Race**			
Black	430 (85.2)	278 (83.7)	152 (87.9)
Other	75 (14.9)	54 (16.3)	21 (12.1)
**Age (years)**	25.1 ± 4.4	24.6 ± 4.4	26.0 ± 4.4
**Marital status**			
Married	152 (30.1)	95 (28.6)	57 (32.9)
Single	353 (69.9)	237 (71.4)	116 (67.1)
**#Katrina traumas**	2.9 ± 2.3	2.9 ± 2.3	3.0 ± 2.1
**Psychological distress**	5.1 ± 4.4	4.9 ± 4.1	5.3 ± 4.1
**BMI**			
Normal	219 (43.4)	152 (45.8)	67 (38.7)
Overweight	140 (27.7)	107 (32.2)	33 (19.1)
Obese	146 (28.9)	73 (22.0)	73 (42.2)
**Self-rated health**			
Excellent	178 (35.3)	120 (36.1)	58 (33.5)
Very good/Good	311 (61.6)	205 (61.7)	106 (61.3)
Fair/Poor	16 (3.2)	7 (2.1)	9 (5.2)

The number of hurricanes experienced was significantly associated with hypertension, with participants exposed to two hurricanes having an adjusted OR of 1.61 (95% CI: 1.00–2.63; *p* = 0.056) and those exposed to three or more hurricanes showing a stronger association (adjusted OR: 1.87, 95% CI: 1.01–3.47; *p* = 0.047) relative to experiencing just one hurricane (table 2). Age was also a significant factor, with a modest but significant increase in hypertension risk per additional year (adjusted OR: 1.07, 95% CI: 1.02–1.12; *p* = 0.003). Obesity was strongly associated with hypertension (adjusted OR: 2.02, 95% CI: 1.29–3.17; *p* = 0.002), while overweight status did not reach statistical significance. Other variables, including race, marital status, number of traumas related to Hurricane Katrina, PD, and self-rated health, did not show significant associations in the adjusted model.

**Table 2.** Logistic regression analysis of factors associated with hypertension (N-505).

Characteristic	Unadjusted OR (95% CI)	*p*	Adjusted OR (95% CI)	*p*
**Hurricanes experienced**				
1	1.00	—	1.00	—
2	1.62 (1.02, 2.58)	0.042	1.61 (1.00, 2.63)	0.056
⩾3	1.88 (1.04, 3.37)	0.035	1.87 (1.01, 3.47)	0.047
**Black Race**	1.41 (0.83, 3.37)	0.218	0.52 (0.86, 2.72)	0.152
**Age (years)**	1.08 (1.03, 1.12)	0.001	1.07 (1.02, 1.12)	0.003
**Married**	1.23 (0.83, 1.82)	0.314	1.22 (0.80, 1.88)	0.357
**# Katrina traumas**	1.02 (0.94, 1.10)	0.629	1.00 (0.91, 1.08)	0.919
**Psychological distress**	1.02 (0.98, 1.07)	0.353	1.02 (0.97, 1.07)	0.513
**BMI**				
Normal	1.00	—	1.00	—
Overweight	0.70 (0.43, 1.14)	0.149	0.66 (0.40, 1.08)	0.097
Obese	2.27 (1.47, 3.50)	<0.001	2.02 (1.29, 3.17)	0.002
**Self-rated health**				
Excellent	1.00	—	1.00	—
Very good/Good	1.07 (0.72, 1.58)	0.736	1.07 (0.71, 1.63)	0.745
Fair/Poor	2.66 (0.94, 7.50)	0.064	2.57 (0.82, 8.00)	0.104

**Figure 1.**
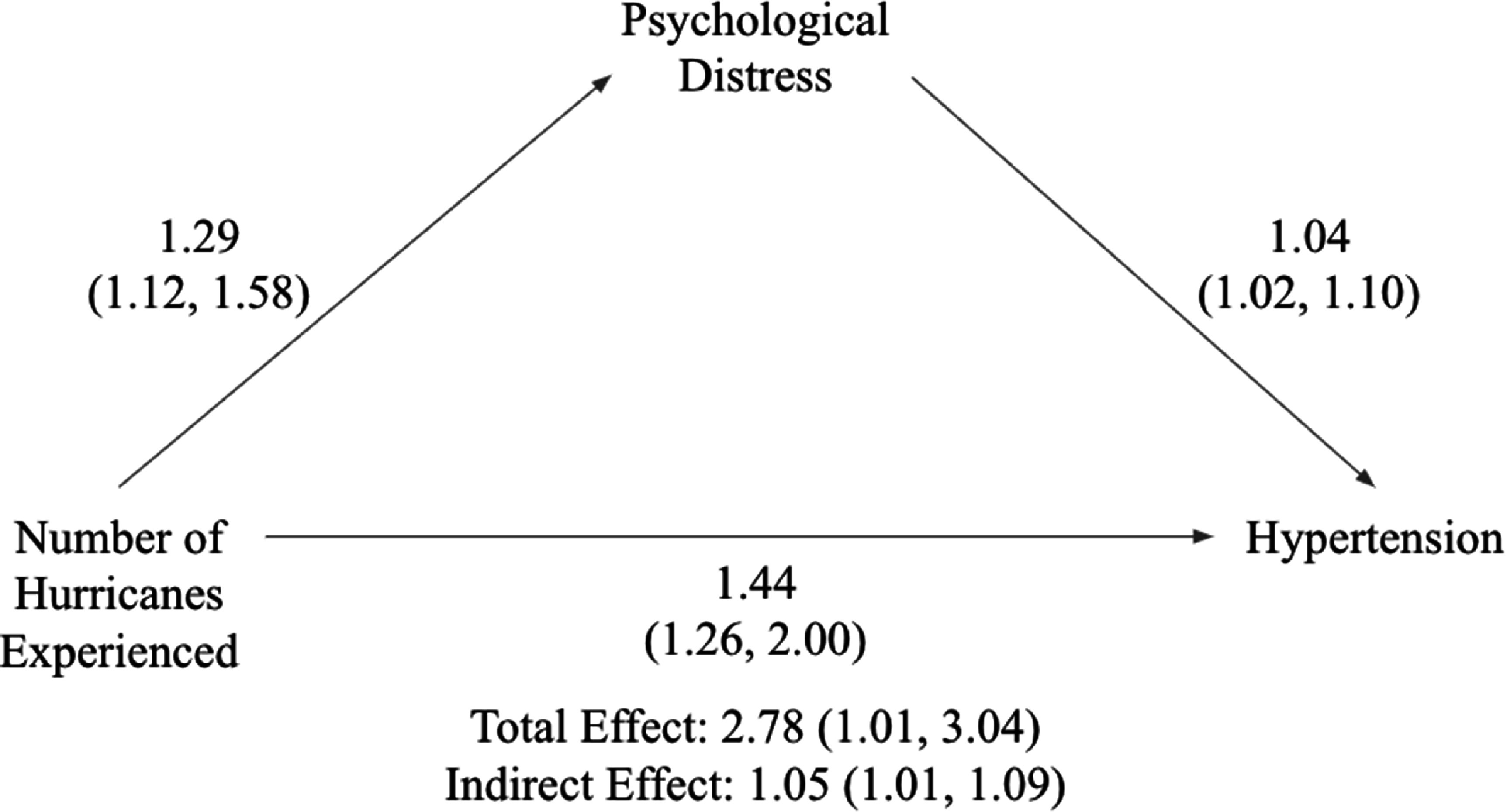
Mediation of hurricane exposure and hypertension by psychological distress.

Figure 1 illustrates a mediation analysis examining the relationship between hurricane exposure and hypertension, with PD assessed at PK2 as the mediator. Directly, hurricane exposure was associated with an increase in the odds of hypertension (OR = 1.44, 95% CI: 1.26, 2.00). Hurricane exposure was also associated with higher PD at PK2 (OR = 1.29, 95% CI: 1.12, 1.58), and higher PD at PK2 further predicted higher odds of hypertension (OR = 1.04, 95% CI: 1.02, 1.10). The indirect effect from hurricane exposure to hypertension via PK2 PD was statistically significant at 1.05 (95% CI: 1.01, 1.09). The total effect of hurricane exposure on hypertension, accounting for both direct and indirect pathways was 2.78 (95% CI: 1.01, 3.04).

## 6. Discussion

Cumulative disaster exposure and hypertension were assessed in a sample of Hurricane Katrina survivors 12 years post-disaster. In our sample of primarily Black mothers who lived in New Orleans at the time of Katrina, we found that hypertension prevalence increased with the number of hurricanes experienced. Our results show that participants who reported a hypertension diagnosis had a higher likelihood of experiencing both two and three or more hurricanes, compared to those without a hypertension diagnosis. Adjusting for sociodemographic factors, clinical measures, potential traumas, and PD, we found that experiencing two hurricanes increased the odds of reporting a hypertension diagnosis, with odds continuing to rise as participants reported experiencing three or more hurricanes, which provides novel evidence that cumulative disaster exposure is associated with adverse physical health conditions.

This finding is consistent with previous studies that report the effects of disaster exposure and cumulative trauma exposure on hypertension [10, 30, 31], but provides a new angle with the additional focus on cumulative disaster exposure. Prior literature has shown an association between disaster exposure and hypertension onset, which is consistent with the current study’s findings [11]. Additionally, results are consistent with the broader trauma literature linking cumulative exposure to adverse physical health outcomes [21]. The link between cumulative disaster exposure and hypertension onset is likely present due to the increased likelihood of chronic stress, which has previously been linked to hypertension [13–15]. As weather-related disasters become more common, it is clear that physical health will be adversely affected for those who live in disaster-prone locations and are likely to have multiple exposures to these disasters throughout their lifetime.

The mediation analysis in this study provides insight into the potential mechanisms through which cumulative disaster exposure might impact physical health, specifically hypertension, among Hurricane Katrina survivors. Our findings suggest an indirect effect from hurricane exposure to hypertension via PD, supporting the notion that the mental health impacts of disaster exposure may contribute to adverse physical outcomes. Participants exposed to multiple hurricanes experienced elevated levels of PD, which in turn was associated with higher odds of hypertension. This pathway underscores the interconnected nature of psychological and physiological responses to chronic stressors such as repeated disaster exposures.

The results align with established literature linking PD and hypertension, providing a potential explanation for the observed association between cumulative disaster exposure and hypertension [17, 32, 33]. Chronic exposure to disasters can lead to sustained psychological stress [7, 9, 34], which has been shown to contribute to long-term dysregulation in physiological systems, including the cardiovascular system. This dysregulation can manifest as elevated blood pressure, ultimately increasing hypertension risk. Our findings add to the understanding of how psychological factors may exacerbate the effects of disaster exposure on physical health, emphasizing the need for a comprehensive approach to disaster response that addresses both mental and physical health.

Furthermore, the total effect of hurricane exposure on hypertension, which includes both direct and indirect pathways, highlights the compounded impact of disaster-related stressors. Even when PD is accounted for, a direct association between hurricane exposure and hypertension remains, suggesting that additional mechanisms may also be at play, potentially including behavioral or environmental factors. For example, repeated disaster exposure may lead to disruptions in healthcare access, medication adherence, or lifestyle changes, all of which can contribute to hypertension risk [35]. These findings underscore the complexity of disaster exposure effects, suggesting that both direct physical impacts and indirect psychological stressors are important contributors to health outcomes in disaster-affected populations.

In light of these results, future research should further explore the role of PD as a mediator in the relationship between disaster exposure and other chronic health conditions. Examining additional pathways, such as behavioral adaptations and healthcare access challenges, could provide a more complete picture of how recurrent disasters impact health. Interventions targeting mental health support following disasters may offer a dual benefit, potentially mitigating both psychological and physical health risks associated with repeated disaster exposures.

Multiple limitations exist within this study. First, these findings may not be generalizable to other disaster survivor populations as the sample was entirely mothers, and mostly Black, unmarried, and low-income at baseline, which is not representative of the general population. However, it is important to note that disasters typically have larger negative effects on racial and ethnic minorities, women, and those who are socio-economically disadvantaged [36]. Second, hypertension prevalence was self-reported, as no medical records were examined. Hypertension is a variable condition that can come and go in a person’s lifetime, which may be difficult to track due to the longitudinal nature of this data. It is likely that cases were missed if participants who had hypertension had entered remission at times of data collection. Additionally, persons with hypertension are often asymptomatic, and diagnoses may have been missed [37], therefore, it is likely that some cases of hypertension are missing from this sample, which may have resulted in smaller odds ratios. It is additionally possible that the women in the sample who reported hypertension, relative to those with the condition but who did not report it, were more advantaged in that they had been screened for hypertension, indicating more healthcare utilization. Finally, hypertension was not assessed at each survey, limiting the number of years of survey data relevant to the analysis.

This study extends prior research by examining the long-term physical impacts of cumulative disaster exposure, with a novel focus on hypertension. Our findings link repeated hurricane exposure in Hurricane Katrina survivors to hypertension diagnosis. This association highlights an urgent need for further research to explore whether other physical health conditions are similarly affected among populations exposed to multiple disasters. Further research should also explore how genetics and family history may impact these results. These findings underscore the profound and lasting health consequences—both physical and mental—of recurrent weather-related disasters.

## Data Availability

The data that support the findings of this study are available upon reasonable request from the authors.
